# Complete genome sequence of a marine *Pseudoalteromonas* bacterial strain

**DOI:** 10.1128/mra.00975-24

**Published:** 2024-12-10

**Authors:** Eric Manirakiza, Timothée Chaumier, Leïla Tirichine

**Affiliations:** 1 Nantes Université, Nantes, France; 2 Institute for Marine and Antarctic Studies (IMAS), Ecology and Biodiversity Centre, University of Tasmania, Hobart, Tasmania, Australia; University of Maryland School of Medicine, Baltimore, Maryland, USA

**Keywords:** *Pseudoalteromonas*, prokaryotes-eukaryotes interactions, active compounds, biofilm

## Abstract

*Pseudoalteromonas* is an abundant bacterial genera, found ubiquitously, including in extreme environments. Its broad metabolic capacity enables unique associations with various organisms. Using PacBio sequencing, we generated the complete genome sequence of a marine *Pseudoalteromonas*, revealing two circular chromosomes and one putative plasmid. The genome data are accessible at https://BacBrowse.univ-nantes.fr.

## ANNOUNCEMENT

*Pseudoalteromonas* species are a group of gamma proteobacteria exclusively isolated from marine waters worldwide (1). These species are often found in extreme environments, including cold habitats and deep-sea sediments (2–4). Interestingly, *Pseudoalteromonas* species are frequently associated with eukaryotic organisms such as sponges, tunicates, and various marine algae, and play a role in controlling harmful dinoflagellate blooms (5–9). This genus forms biofilm and produces active compounds (10, 11). The ecological success and diverse associations of this genus reveal its potential as a vast reservoir of active metabolites, making it highly relevant for both fundamental and applied research. The genus *Pseudoalteromonas* is classified into pigmented and non-pigmented clades, with pigmented species often linked to a higher propensity for natural product synthesis (12–14). Despite the identification of over 2,000 species, the full extent of the genus diversity remains largely unexplored.

**Fig 1:**
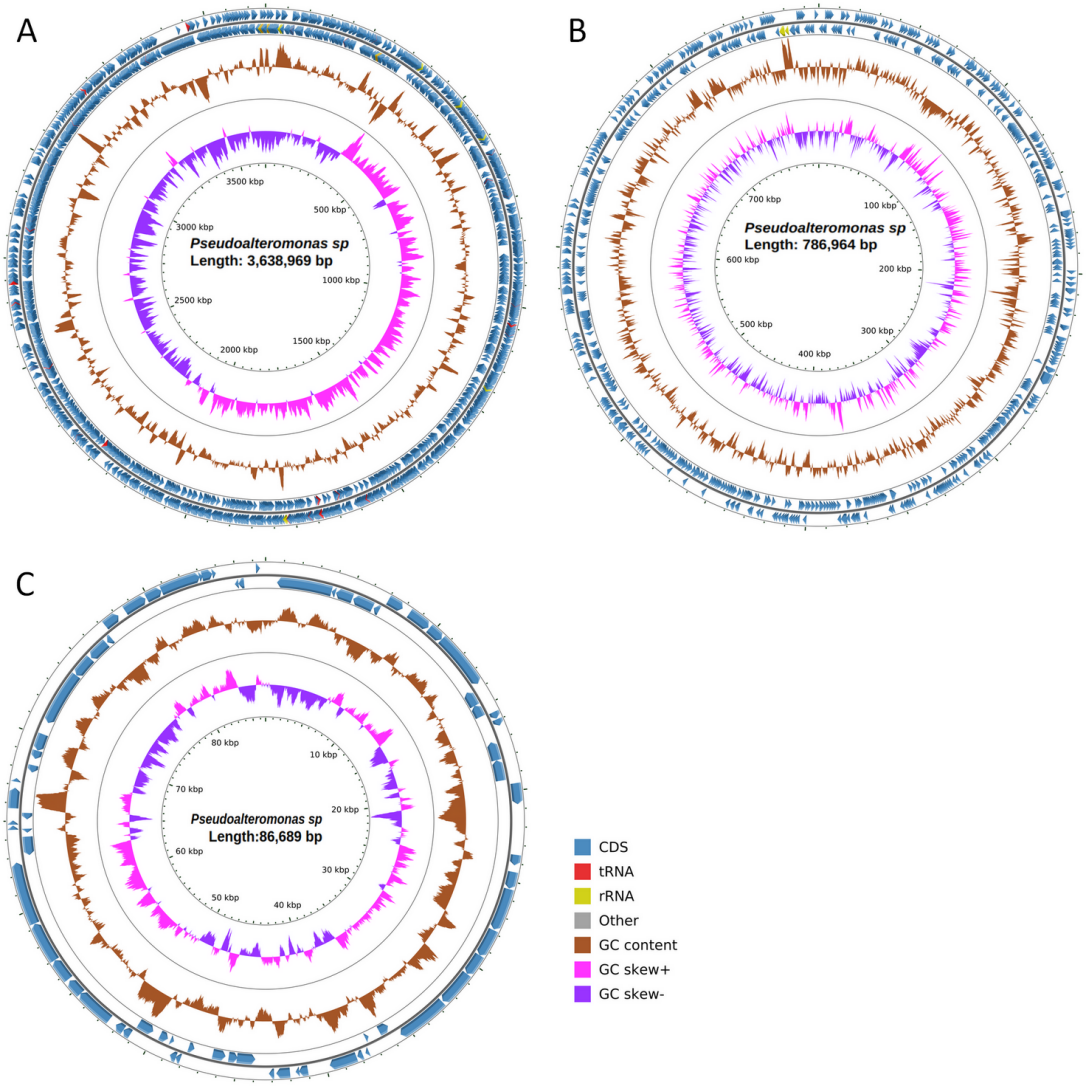
Graphical circular map of the large chromosome (**A**), small chromosome (**B**), and plasmid (**C**) of *Pseudoalteromonas* sp. Blue arrows represent coding sequences. GC content is shown in brown (values outside the circle represent GC content that deviates from the average, either higher or lower). tRNA and rRNA are represented in red and yellow, respectively. GC skew is depicted in pink to represent positive values and in purple to represent negative values.

Here, we report the complete genome sequences of a pigmented *Pseudoalteromonas* sourced from the National Collection of Industrial Microorganisms (NCIMB_1079) as *Pseudomonas fluorescens*. This discrepancy has been reported to NCIMB. *Pseudoalteromonas* (1,079 NCIMB) was isolated on 12 October 1962, from sea water, 16 km East South East of Aberdeen in the United Kingdom (latitude 57°6′15″N, longitude 1°44′15″W). The salinity of sea water at the sampling site was 34.7‰ (15). Upon isolation, the strain was plated on Anderson’s marine medium (16). Upon purchase in October 2019, the strain was stored as a glycerol stock at −80°C. Cells were cultured in liquid Luria–Bertani (LB) medium at 30°C with shaking at 180 rpm for 48 h. DNA was extracted using the MagAttract High Molecular Weight DNA Kit (Cat. no. 67563) following the manufacturer’s instructions. Genomic DNA was sheared with Covaris g-TUBES at 4,000 RPM. The DNA libraries were prepared without size selection using SMRTbell Express Template Prep Kit 2.0 protocol following the Pacific Biosciences Procedure. DNA was sequenced by Genome Quebec using the Pacific Biosciences Sequel platform with Circular Consensus Sequencing (CCS). SmrtLink v8.0 was used to monitor run QC, demultiplex reads, remove adapter sequences, and generate subreads filtered for minimum read quality. CCS reads were generated from subreads with CCS 6.4.0 and assembled with Canu (v2.2) (17). Genome coverage was 118.11×, with an estimated size of 4.5 Mb (Table 1, Fig. 1). Among the three contigs, two were identified as chromosomes and the third as a plasmid. The large chromosome, small chromosome, and plasmid display overlaps at their ends measuring 15.4, 14.2, and 16.4 kb, respectively. The identification of plasmids was performed using two plasmid prediction tools: PlasmidHunter (v1.4) (18) and PLASMe (v1.1) (19). These tools predicted a plasmid with a length of 86,689 bp (Fig. 1). Gene annotation and functional assignment were performed using anvi'o v7.1 (20). Additional gene annotations were performed with DFAST 1.2.20 (21), Prokka (v1.14.6) (22) with compliant parameter and EggNOG (v2.1.12) (23). For all softwares, default parameters were used except where otherwise noted. The parameters -m [diamond] and pfam_realign [realign] were used for EggNOG. The assembled genome is available on BacBrowse (https://BacBrowse.univ-nantes.fr).

**TABLE 1:** Genome features of *Pseudoalteromonas* bacterial strain

	Pseudoalteromonas
Number of subreads	1,472,349
*N*_50_ of subreads	7,745
Number of CCS reads	76,463
*N*_50_ of CCS reads (bp)	8,902
Average length of CCS reads	7,015.55
Genome size (bp)	4,512,622
Number of contigs	3
Mean genome coverage	118.11×
Contamination^a^	0
Completness (%)^*a*^	100
GC content (%)	39.4
Coding region (%)	88.1
Average length CR (bp)	977.47
Intergenic region (%)	12.2
Average length IR (bp)	168.08
Total repeats	231
Total tRNAs	106
Total rRNAs	25
Total pseudogenes	436

^*a*^ Genome contamination and completeness were determined using CheckM (24).

## Data Availability

The genome sequences of *Pseudoalteromonas* sp., has been deposited in GenBank under the Bioproject PRJNA1101843 .The genome assembly and sequencing raw reads have been deposited in the NCBI Sequence Read Archive under accession numbers ASM4056769v1 and SRR28815868, respectively.

## References

[1] Enger O, Nygaard H, Solberg M, Schei G, Nielsen J, Dundas I. 1987. Characterization of Alteromonas denitrificans sp. nov. Int J Syst Bacteriol 37:416–421. doi:10.1099/00207713-37-4-416

[2] Parrilli E, Tedesco P, Fondi M, Tutino ML, Lo Giudice A, de Pascale D, Fani R. 2021. The art of adapting to extreme environments: the model system Pseudoalteromonas. Phys Life Rev 36:137–161. doi:10.1016/j.plrev.2019.04.003 31072789

[3] Qin Q-L, Li Y, Zhang Y-J, Zhou Z-M, Zhang W-X, Chen X-L, Zhang X-Y, Zhou B-C, Wang L, Zhang Y-Z. 2011. Comparative genomics reveals a deep-sea sediment-adapted life style of Pseudoalteromonas sp. SM9913. ISME J 5:274–284. doi:10.1038/ismej.2010.103 20703316 PMC3105692

[4] Bian F, Xie B-B, Qin Q-L, Shu Y-L, Zhang X-Y, Yu Y, Chen B, Chen X-L, Zhou B-C, Zhang Y-Z. 2012. Genome sequences of six Pseudoalteromonas strains isolated from Arctic sea ice. J Bacteriol 194:908–909. doi:10.1128/JB.06427-11 22275105 PMC3272954

[5] Ivanova EP, Kiprianova EA, Mikhailov VV, Levanova GF, Garagulya AD, Gorshkova NM, Yumoto N, Yoshikawa S. 1996. Characterization and identification of marine Alteromonas nigrifaciens strains and emendation of the description. Int J Syst Bacteriol 46:223–228. doi:10.1099/00207713-46-1-223 9542094

[6] Ivanova EP, Kiprianova EA, Mikhailov VV, Levanova GF, Garagulya AD, Gorshkova NM, Vysotskii MV, Nicolau DV, Yumoto N, Taguchi T, Yoshikawa S. 1998. Phenotypic diversity of Pseudoalteromonas citrea from different marine habitats and emendation of the description. Int J Syst Bacteriol 48:247–256. doi:10.1099/00207713-48-1-247 9542094

[7] Holmström C, James S, Neilan BA, White DC, Kjelleberg S. 1998. Pseudoalteromonas tunicata sp. nov.,a bacterium that produces antifouling agents. Int J Syst Bacteriol 48:1205–1212. doi:10.1099/00207713-48-4-1205 9828422

[8] Egan S, Thomas T, Holmström C, Kjelleberg S. 2000. Phylogenetic relationship and antifouling activity of bacterial epiphytes from the marine alga Ulva lactuca. Environ Microbiol 2:343–347. doi:10.1046/j.1462-2920.2000.00107.x 11200436

[9] Lovejoy C, Bowman JP, Hallegraeff GM. 1998. Algicidal effects of a novel marine Pseudoalteromonas isolate (class proteobacteria, gamma subdivision) on harmful algal bloom species of the genera Chattonella, Gymnodinium, and Heterosigma. Appl Environ Microbiol 64:2806–2813. doi:10.1128/AEM.64.8.2806-2813.1998 9687434 PMC106776

[10] Casillo A, Lanzetta R, Parrilli M, Corsaro MM. 2018. Exopolysaccharides from marine and marine extremophilic bacteria: structures, properties, ecological roles and applications. Mar Drugs 16:69. doi:10.3390/md16020069 29461505 PMC5852497

[11] Skovhus TL, Ramsing NB, Holmström C, Kjelleberg S, Dahllöf I. 2004. Real-time quantitative PCR for assessment of abundance of Pseudoalteromonas species in marine samples. Appl Environ Microbiol 70:2373–2382. doi:10.1128/AEM.70.4.2373-2382.2004 15066834 PMC383141

[12] Egan S, James S, Holmström C, Kjelleberg S. 2002. Correlation between pigmentation and antifouling compounds produced by Pseudoalteromonas tunicata. Environ Microbiol 4:433–442. doi:10.1046/j.1462-2920.2002.00322.x 12153584

[13] Holmström C, Kjelleberg S. 1999. Marine Pseudoalteromonas species are associated with higher organisms and produce biologically active extracellular agents. FEMS Microbiol Ecol 30:285–293. doi:10.1111/j.1574-6941.1999.tb00656.x 10568837

[14] Bowman JP. 2007. Bioactive compound synthetic capacity and ecological significance of marine bacterial genus pseudoalteromonas. Mar Drugs 5:220–241. doi:10.3390/md504220 18463726 PMC2365693

[15] A. JIW. 1962. Heterotrohpic bacteria of North sea water, Glasgow

[16] Brown AE. 2019. Benson's microbiological applications: laboratory manual in general microbiology. In T. E. M.-H. Education

[17] Koren S, Walenz BP, Berlin K, Miller JR, Bergman NH, Phillippy AM. 2017. Canu: scalable and accurate long-read assembly via adaptive k-mer weighting and repeat separation. Genome Res 27:722–736. doi:10.1101/gr.215087.116 28298431 PMC5411767

[18] Tian R, Imanian B. 2023. PlasmidHunter: accurate and fast prediction of plasmid sequences using gene content profile and machine learning. Bioinformatics. doi:10.1101/2023.02.01.526640 PMC11770376 38960405

[19] Tang X, Shang J, Ji Y, Sun Y. 2023. PLASMe: a tool to identify PLASMid contigs from short-read assemblies using transformer. Nucleic Acids Res 51:e83. doi:10.1093/nar/gkad578 37427782 PMC10450166

[20] Eren AM, Kiefl E, Shaiber A, Veseli I, Miller SE, Schechter MS, Fink I, Pan JN, Yousef M, Fogarty EC, et al.. 2021. Community-led, integrated, reproducible multi-omics with anvi’o. Nat Microbiol 6:3–6. doi:10.1038/s41564-020-00834-3 33349678 PMC8116326

[21] Tanizawa Y, Fujisawa T, Kaminuma E, Nakamura Y, Arita M. 2016. DFAST and DAGA: web-based integrated genome annotation tools and resources. Biosci Microbiota Food Health 35:173–184. doi:10.12938/bmfh.16-003 27867804 PMC5107635

[22] Seemann T. 2014. Prokka: rapid prokaryotic genome annotation. Bioinformatics 30:2068–2069. doi:10.1093/bioinformatics/btu153 24642063

[23] Cantalapiedra CP, Hernández-Plaza A, Letunic I, Bork P, Huerta-Cepas J. 2021. eggNOG-mapper v2: functional annotation, orthology assignments, and domain prediction at the metagenomic scale. Bioinformatics. doi:10.1101/2021.06.03.446934 PMC8662613 34597405

[24] Parks DH, Imelfort M, Skennerton CT, Hugenholtz P, Tyson GW. 2015. CheckM: assessing the quality of microbial genomes recovered from isolates, single cells, and metagenomes. Genome Res 25:1043–1055. doi:10.1101/gr.186072.114 25977477 PMC4484387

